# Interaction between gut microbiota and immunity in health and intestinal disease

**DOI:** 10.3389/fimmu.2025.1673852

**Published:** 2025-11-10

**Authors:** Qishang Wang, Qingguo Meng, Yuchao Chen, Yaxin Liu, Xinru Li, Jinjie Zhou, Yuchu Ma, Zihan Yu, Xin Chen

**Affiliations:** 1 Department of Gastroenterology and Hepatology, Tianjin Medical University General Hospital, Tianjin, China; 2 Tianjin Institute of Digestive Disease, Tianjin Medical University General Hospital, Tianjin, China

**Keywords:** gut microbiota, immune homeostasis, adaptive and innate immunity, dysbiosis, intestinal disease

## Abstract

The gut microbiota plays a fundamental role in establishing and maintaining host immune homeostasis through dynamic, bidirectional interactions with the innate and adaptive immune systems. This review synthesizes current knowledge on how commensal microbes guide the development and function of the intestinal immune system. Conversely, we examine how the host immune system, including immunoglobulin A (IgA) and T-cell responses, actively shapes microbial composition and colonization resistance. Disruptions in this equilibrium (dysbiosis) are critically implicated in pathogenesis. We explore the dysbiosis-immune axis in inflammatory bowel diseases (IBD), irritable bowel syndrome (IBS), and colorectal cancer (CRC), highlighting how specific microbial taxa and their metabolites influence disease progression through immune modulation. Furthermore, we discuss how acute infectious insults model the breakdown of this mutualism.

## 1 introduction

The mammalian organism hosts an extensive consortium of microorganisms across epithelial surfaces, notably within gastrointestinal, cutaneous, and mucosal niches, collectively designated as the microbiome (1). Among these sites, the intestinal tract sustains the highest microbial density and phylogenetic diversity relative to other host compartments (2). Inhabiting the human gut are approximately 1,500 bacterial species and 100 trillion microbial cells, whose collective genome encodes over tenfold more unique genes than the human genome (3, 4). Contemporary research underscores that gut microbiota dynamically modulates essential host physiological processes—spanning circadian regulation, nutrient assimilation, metabolic pathways, and immune function—rather than operating as inert residents (5, 6).

This host-microbiota interface represents an evolutionary co-adaptation spanning millennia, fundamentally characterized by mutualistic symbiosis (7). Ecologically, mammals and commensal microorganisms have co-evolved to establish homeostatic equilibrium (8). Through persistent immunological stimulation, the complex gut microbial community critically orchestrates the development and functional maturation of both innate and adaptive immunity (9, 10). In physiological states, microbiota-derived metabolites—generated via anaerobic fermentation of dietary substrates and host-microbial co-metabolites—serve as key immunomodulatory messengers (11). Specifically, short-chain fatty acids (SCFAs) and related compounds directly interface with host cellular receptors to fine-tune immune responses (12). The gut immune system reciprocally influences the gut microbiota. It establishes immune tolerance toward commensal and harmless microorganisms while maintaining effective immune responses against pathogenic infections. Under healthy conditions, this host immune response to the intestinal microbiota is strictly compartmentalized to the mucosal surface (13). Therefore, the gut microbiota and the host immune system exhibit significant bidirectional regulation.

However, this meticulously balanced host-microbiota symbiosis is vulnerable to disruption. Pathological alterations in the gut microbial ecosystem, termed dysbiosis, are increasingly recognized as critical factors in the pathogenesis of a spectrum of intestinal disorders (14–17). Critically, immune dysregulation constitutes a central mechanistic link underpinning these conditions. Mounting evidence positions the gut microbiome as a pivotal regulator of immune ontogeny, response calibration, and mucosal barrier integrity (18). Consequently, disruptions in the intricate dialogue between the microbiota and the host immune system—encompassing failures in tolerance induction, aberrant inflammatory responses, and compromised barrier function—are implicated in the initiation and perpetuation of chronic inflammation observed in diseases such as IBD, IBS, and CRC. In this review, we synthesize current mechanistic insights into this bidirectional microbiota-immune crosstalk across both physiological homeostasis and major intestinal pathologies. Furthermore, we evaluate emerging therapeutic strategies aimed at modulating this critical axis to restore health, highlighting the pivotal role of understanding these interactions for developing novel interventions against dysbiosis-associated intestinal diseases.

## 2 Microbiota-immune interplay in physiological conditions

### 2.1 Microbiota-driven maturation and maintenance of intestinal immunity

Early colonization of mucosal surfaces in mammalian hosts plays a decisive role in immune system maturation (19). While debate continues regarding prenatal microbial exposure *in utero*, overwhelming evidence confirms a substantial microbial influx immediately after birth, predominantly derived from maternal microbiota (20, 21). Delivery mode critically shapes initial microbial composition: vaginally delivered infants acquire microbes resembling maternal vaginal/enteric communities (e.g., *Lactobacillus, Prevotella*), whereas cesarean-delivered neonates are colonized by skin-associated taxa (e.g., *Staphylococcus, Corynebacterium*) (22). Beyond providing passive immunity through maternal antibodies, breast milk induces commensal microbiota-dependent protective immunity, as demonstrated in germ-free models (18, 20).

The first three years of life constitute a critical developmental window characterized by high microbiota volatility preceding stabilization into adult-like configurations (23–25). This plasticity increases vulnerability to environmental microbial perturbations that may disrupt immunoregulatory circuits with lifelong consequences (26). Early-life microbial colonization limits the expansion of invariant natural killer T (iNKT) cells, in part via production of sphingolipids, to prevent potential disease-promoting activity within the intestinal lamina propria and the lungs (27). Neonatal immune immaturity manifests clinically as heightened pathogen susceptibility – with infectious diseases representing leading causes of childhood mortality – and dysregulated inflammation, exemplified by necrotizing enterocolitis (NEC) in preterm infants (28–30).

Studies using germ-free mice reveal mechanistic insights into microbiota-driven immune maturation. These microbiome-deficient animals exhibit multiple immunological deficits: impaired development of gut-associated lymphoid tissues; 30% reduction in αβ/γδ intraepithelial lymphocytes; absence of intestinal lamina propria Th17 cells (inducible by segmented filamentous bacteria colonization); diminished Th1 responses compromising intracellular pathogen clearance; and markedly reduced IgA secretion – all reversible upon microbial colonization (31–37).

Collectively, early-life microbial colonization orchestrates immune development through three interconnected pathways: 1) Initial seeding mechanisms (birth mode and feeding) establish foundational microbiota architecture; 2) Dynamic microbiota-immune crosstalk during the plasticity window of less than 3 years directs tolerance programming; 3) Microbial antigens directly drive lymphoid tissue maturation and T-cell differentiation. Disruption of this process predisposes to either immunodeficiency or pathological inflammation, underscoring the necessity of maintaining early microbiota homeostasis for lifelong immune competence. germ-free animal models remain indispensable for mechanistic dissection, though future research must address strain-specific functions and clinical translation challenges.

Crucially, the influence of the gut microbiota on immune function extends far beyond early development and persists throughout adulthood in healthy individuals. In the mature gut, the established commensal community plays an indispensable role in the continuous maintenance, education, and fine-tuning of the immune system.

Perpetual Antigenic Stimulation & Immune Training: The diverse microbial antigens provide constant, low-level stimulation to the intestinal immune system. This persistent exposure is essential for maintaining the pool and functionality of resident immune cells, including lamina propria lymphocytes (e.g., Th17, Tregs, IELs), innate lymphoid cells (ILCs), and antigen-presenting cells (APCs) like dendritic cells and macrophages. It trains these cells to distinguish between commensals and pathogens, reinforcing immune tolerance towards the former while preserving vigilance against the latter (38, 39).

Metabolite-Mediated Immunomodulation: Microbiota-derived metabolites, particularly SCFAs like butyrate, propionate, and acetate, remain critical immunomodulatory messengers in the adult gut. SCFAs signal through G-protein-coupled receptors (GPR41, GPR43, GPR109a) and inhibit histone deacetylases (HDACs) in various immune cells. This promotes anti-inflammatory responses, enhances epithelial barrier integrity, drives the differentiation and function of regulatory T cells (Tregs), and modulates macrophage and dendritic cell function towards a tolerant phenotype (40–42).

Maintenance of Barrier Surveillance and IgA Dynamics: The healthy adult microbiota continuously stimulates IgA production by plasma cells in the gut-associated lymphoid tissue (GALT). IgA, particularly secretory IgA (sIgA), plays a vital role in coating commensal bacteria, restricting their penetration into the epithelium and lamina propria, shaping microbial composition, and neutralizing potential pathobionts. This dynamic IgA coating is a hallmark of adult immune-microbiota mutualism (43, 44).

Sustaining Innate Effector Functions: Commensals continue to prime systemic innate immunity in adults. For example, microbial components (e.g., peptidoglycan fragments detected by NOD1) enhance neutrophil bone marrow egress and functional readiness. Microbiota-derived signals also maintain the “inflammatory anergy” of intestinal macrophages, preventing inappropriate activation against commensals (45–47).

Adaptation to Environmental Changes: The adult microbiota-immune axis allows for adaptation. Changes in diet, transient pathogen exposure, or mild stressors can induce shifts in microbial composition and metabolite profiles. A well-established immune system, calibrated by the microbiota, can dynamically respond to these shifts, restoring homeostasis without triggering chronic inflammation (48).

Therefore, the adult gut microbiota is not merely a passive resident but an active participant in a continuous, bidirectional crosstalk essential for sustaining immune quiescence, barrier defense, and the capacity for appropriate inflammatory responses throughout life. Disruption of this mature equilibrium (dysbiosis) can lead to immune dysfunction and contribute to disease pathogenesis, as discussed in subsequent sections.

### 2.2 Interactions between the innate immune system and the microbiota

**Figure 1:**
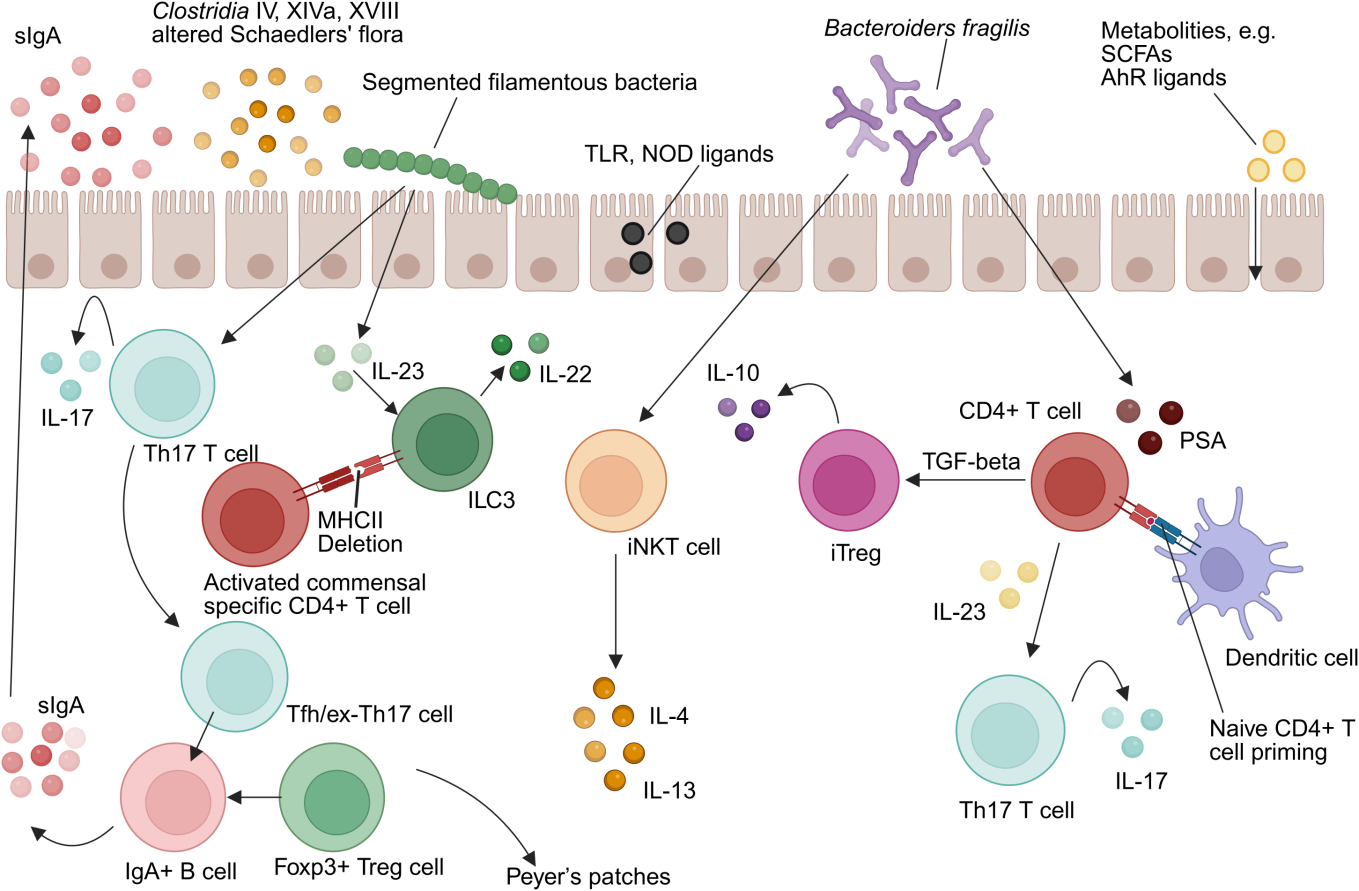
Intestinal microbiota-immunity interplay in homeostasis. TLR, Toll-like receptor; NOD, Nucleotide-binding oligomerization domain-containing protein; IL-10, Interleukin-10; IL-17, Interleukin-17; IL-4, Interleukin-4; IL-13, Interleukin-13; IL-22, Interleukin-22; IL-23, Interleukin-23; ILC3, group 3 innate lymphoid cell; Th17, T helper 17 cell; MHCII, Major histocompatibility complex class II; Treg, Regulatory T cell; iNKT, Invariant natural killer T cell; TGF-β, Transforming growth factor beta; iTregs, Induced regulatory T cell; SCFAs, Short-chain fatty acids; AhR, Aryl hydrocarbon receptor; PSA, Polysaccharide A; DC, Dendritic cell (Created in https://BioRender.com).

The gut microbiota critically shapes innate immunity through multifaceted molecular dialogues involving both local mucosal environments and systemic immune compartments (**Figure 1**). Microbial-derived ligands—including Toll-like receptor (TLR) and nucleotide-binding oligomerization domain (NOD) agonists—as well as immunomodulatory metabolites such as SCFAs and aryl hydrocarbon receptor (AhR) ligands, engage host pattern recognition receptors (PRRs) to fine-tune immune activation and maintain homeostasis (49).

#### 2.2.1 Mucosal programming of APCs

Commensal organisms have co-evolved with intestinal immune cells to promote immunological tolerance while preserving responsiveness to pathogens. Dendritic cells (DCs) in Peyer’s patches, for example, secrete significantly higher levels of interleukin-10 (IL-10) relative to splenic DCs under comparable stimuli, reflecting niche-specific tolerogenic adaptation (50). Intestinal macrophages exhibit a unique phenotype termed “inflammatory anergy,” whereby pro-inflammatory cytokine secretion (e.g., TNF-α, IL-6) is suppressed even in the presence of TLR ligands (45–47). This regulatory phenotype is microbiota-dependent and essential for sustaining mucosal tolerance (37). Furthermore, group 3 ILC(ILC3s) express MHC class II molecules that suppress activation of commensal-specific CD4^+^ T cells, serving as a checkpoint to prevent aberrant immune responses (51).

Mechanistically, microbiota-derived ATP activates P2X receptors on CD70^+^ intestinal DCs, promoting differentiation of RORγt^+^ Th17 cells via IL-6 and IL-23 pathways (52, 53). Germ-free models reinforce this dependence: monocolonization with *Escherichia coli* restores depleted intestinal DC populations without affecting systemic counterparts (37).

#### 2.2.2 Systemic modulation of innate effector cells

Beyond the intestinal milieu, the microbiota exerts systemic effects on innate immunity. Germ-free mice display pronounced neutropenia, with a 30–40% reduction in circulating neutrophils, accompanied by compromised phagocytic activity and diminished production of reactive oxygen and nitrogen species (54–57). These defects are only partially reversed upon recolonization, indicating critical windows for microbial imprinting.

A pivotal role is played by NOD1, a cytosolic PRR that recognizes peptidoglycan fragments from Gram-negative bacteria. NOD1 activation enhances neutrophil myeloperoxidase activity and promotes their egress from bone marrow, effectively linking gut microbial sensing to peripheral immune readiness (58). Additionally, the microbiota influences natural killer (NK) cell cytotoxicity and mast cell protease expression, although the underlying pathways remain incompletely elucidated (59–61).

#### 2.2.3 PRRs as regulators of microbiota composition

Host PRRs not only respond to microbial cues but actively modulate microbial ecology. Mice deficient in TLR5 exhibit significant shifts in microbiota composition—specifically, expansion of Proteobacteria and reduction in Bacteroidetes—alongside increased susceptibility to colitis, obesity, and metabolic syndrome (62–65). Similar dysbiosis has been observed in NOD1-, NOD2-, and NLRP6-deficient models, implicating cytosolic and inflammasome-associated PRRs in maintaining microbial homeostasis (66–69).

Notably, inflammasome dysfunction—particularly NLRP6 deficiency—results in transmissible dysbiosis mediated by impaired goblet cell mucin secretion and reduced antimicrobial peptides (AMPs) expression. Such microbiota shifts can propagate metabolic and inflammatory disorders to co-housed wild-type animals (69).

This collective evidence underscores a crucial paradigm: the innate immune system is not a passive sensor but an active architect of the gut microbial environment. Through the continuous expression of PRRs and effector molecules like AMPs, the host shapes the taxonomic composition and functional potential of the microbiota, determining which species can thrive in the intestinal niche. This active sculpting prevents the expansion of pro-inflammatory taxa and enforces a homeostatic community structure that is mutually beneficial. Thus, the dialogue between innate immunity and the microbiota is fundamentally bidirectional; innate signals educate the immune system, while immune mechanisms, in turn, mold the microbiota.

#### 2.2.4 AMPs and microbial spatial organization

Paneth cells in the intestinal crypts secrete α-defensins and RegIIIγ, which constitute essential components of the mucosal chemical barrier. These AMPs maintain the sterility of the inner mucus layer and prevent microbial encroachment. Deficiency in these peptides disrupts spatial segregation, resulting in bacterial translocation into epithelial niches and subsequent epithelial hyperplasia via TLR signaling (70–73). Similarly, structural defects in the mucus layer compromise barrier integrity and foster pro-inflammatory microbial shifts (74).

In summary, microbiota-derived signals program innate immunity through PRR-mediated tolerance induction, systemic effector priming, and AMPs-dependent spatial containment. Disruption of this intricate crosstalk establishes a permissive environment for metabolic and inflammatory disorders.

### 2.3 Crosstalk between the adaptive immune system and the microbiota

The adaptive immune system has co-evolved with the gut microbiota to establish a highly specialized and reciprocal relationship essential for maintaining intestinal homeostasis. This mutualistic interaction is evident from studies showing pronounced microbial dysbiosis in immunodeficient mice lacking functional T or B cells (75). Two principal immunological axes underlie this crosstalk: T cell–mediated regulation of microbial composition and secretory IgA–dependent maintenance of mucosal equilibrium, with T cells playing a dominant role in shaping microbiota configuration (76). It is increasingly clear that the adaptive immune system exerts profound selective pressure on the microbiota, effectively functioning as a sophisticated ecological filter that determines microbial fitness and enforces community stability.

#### 2.3.1 CD4^+^ T cell subset differentiation orchestrated by commensals

Within the intestinal microenvironment, microbial antigens direct the lineage commitment of naïve CD4^+^ T cells into distinct functional subsets—including Th1, Th2, Th17, and Tregs—thereby sculpting immune tone and microbial tolerance. Conversely, the resulting cytokine milieu actively feeds back to shape the microbial landscape. For instance, the IL-17 and IL-22 produced by Th17 cells stimulate epithelial cells to secrete AMPs, which directly target specific bacteria and influence community assembly. Similarly, the anti-inflammatory cytokines like IL-10 and TGF-β derived from Tregs promote a tolerogenic environment that favors the persistence of beneficial, anti-inflammatory commensals. This creates a self-reinforcing loop where microbes induce specific T cell responses, which then modify the environment to favor or suppress different microbial groups.

Th1/Th2 Axis Regulation: Germ-free mice exhibit a Th2-skewed cytokine milieu, typified by elevated IL-4 and IL-5 levels, which correlates with increased susceptibility to allergic diseases such as asthma and eczema (77–79). Colonization with *Bacteroides fragilis*, through its capsular polysaccharide A (PSA), re-establishes Th1/Th2 equilibrium. PSA is internalized by lamina propria dendritic cells via a TLR2-dependent pathway, leading to the differentiation of naïve CD4^+^ T cells into IL-10–secreting inducible Tregs (iTregs) or, in the presence of IL-23, Th17 cells (80).

Functional Dichotomy of Th17 Cells: Specific microbiota members shape distinct Th17 phenotypes. Segmented filamentous bacteria (SFB) elicit homeostatic, non-pathogenic Th17 cells, whereas pathogens such as *Citrobacter rodentium* induce inflammatory Th17 responses (81, 82). Mechanistically, SFB colonization activates the ILC3–IL-22–SAA axis, promoting IL-17A expression in RORγt^+^ Th17 cells (81). Although SFB is scarcely detected in the human microbiome, other taxa such as *Eggerthella lenta* may assume equivalent immunomodulatory roles (83).

Tregs Expansion and Function: *Clostridium clusters* IV and XIVa promote colonic Tregs accumulation via SCFAs production, while PSA from *B. fragilis* signals through TLR2 to suppress Th17-driven inflammation (84, 85). Colonic Tregs often bear microbiota-reactive TCRs and low Helios expression, indicating peripheral induction. Moreover, T follicular helper (Tfh) and exTh17 cells in Peyer’s patches contribute to B cell class-switch recombination and sIgA production, reinforcing microbiota compartmentalization and compositional control (86, 87).

#### 2.3.2 IgA-mediated regulation of mucosal microbial ecology

SIgA represents a critical effector of adaptive immunity at mucosal surfaces, mediating immune exclusion, neutralization, and microbial homeostasis. The functional relevance of IgA is underscored by its dependency on T cell help for class switching and antigen specificity. Crucially, IgA is a principal mechanism by which the host actively and continuously shapes the gut microbiota. Rather than simply neutralizing pathogens, IgA imposes a selective pressure that governs bacterial gene expression, metabolic activity, and spatial distribution within the gut lumen.

T Cell–Dependent IgA Induction: In T cell–deficient models, Tregs are essential for restoring microbiota-reactive IgA responses (e.g., against flagellin), while Th17 cells promote antigen-specific IgA during mucosal perturbations (88–90). SFB uniquely co-induces both Th17 differentiation and IgA coating (91, 92). Germ-free mice require colonization with ≥10^9^ CFU of viable bacteria to initiate robust IgA secretion (34).

Mechanisms of IgA-Mediated Homeostasis: IgA enforces microbial balance via several mechanisms. In AID^-^/^-^ mice, which are deficient in class-switch recombination, SFB overgrowth can be reversed by IgA reconstitution (93, 94). Flagellin-specific IgA dampens bacterial flagellin expression, thereby reducing TLR5 activation and intestinal inflammation (95). Additionally, IgA suppresses Proteobacteria expansion during neonatal microbiota development (Mirpuri et al., 2014). Disruption of T cell–dependent IgA responses (e.g., via MyD88 deletion in T cells) alters IgA coating patterns, resulting in dysbiosis (96–98). High-affinity IgA not only immobilizes luminal microbes to prevent epithelial contact but also preserves microbiota diversity (87). This dynamic, antigen-specific selection is a quintessential example of the host immune system actively gardening its microbial inhabitants. The IgA repertoire adapts to the current microbial residents, and in doing so, it modulates their behavior and abundance, preventing the overdominance of any single strain and maintaining a diverse, stable ecosystem. This process is a continuous and active negotiation between the host and its microbiota.

Source and Distribution of IgA: GALT are rich in IgA^+^ plasma cells, which secrete up to 0.8 grams of IgA per meter of intestine per day (99). Germ-free mice exhibit marked reductions in both IgA-producing cells and intestinal IgA levels, as well as defective germinal center formation in the spleen (100, 101). They also show skewed immunoglobulin profiles, including elevated IgE and diminished IgG, indicative of a Th2-biased systemic state (102, 103). While BCR diversity is shaped by microbial exposure, the mechanisms underlying selective commensal tolerance remain incompletely understood (104).

#### 2.3.3 Beyond CD4^+^ T cells and IgA: additional adaptive immune modulators

Although IgA and CD4^+^ T cells are central to microbiota-host dialogue, other adaptive immune components are also involved. For instance, CD8^+^ intraepithelial lymphocytes (IELs), enriched in the gut epithelium, require microbial signals for functional competence and homeostasis. In germ-free mice, impaired clonal expansion of CD8^+^ IELs leads to diminished cytotoxic potential, compromising mucosal immunity (105). These IELs influence peripheral compartments, including marginal zone B cells and plasmacytoid dendritic cells (106).

In gut mucosal immunity, besides secretory sIgA, other immunoglobulins—IgG, IgE, and IgM—also contribute to immune regulation. IgG enters the lumen during barrier disruption (e.g., IBD), opsonizing microbes, activating complement, and driving inflammation, while also serving as a disease biomarker. IgE, low in healthy gut, can trigger mast cell degranulation in allergies and IBS, promoting hypersensitivity and dysmotility. IgM acts as a first-line defense and compensates for IgA deficiency via secretory IgM (sIgM), agglutinating microbes and moderating dysbiosis, though it cannot fully substitute IgA’s anti-inflammatory functions. Together, these antibodies coordinate to maintain intestinal immune homeostasis and contribute to pathology when dysregulated.

iNKT cells represent another subset regulated by the microbiota. Germ-free mice show iNKT immaturity and hypo-responsiveness, which can be reversed through colonization with *B. fragilis* or administration of microbial sphingolipids. These interventions enhance iNKT development and confer resistance to colitis (107).

In general, these findings underscore the intricate and dynamic interplay between the adaptive immune system and the gut microbiota. CD4^+^ T cell subsets—particularly Th17 and Tregs—respond to distinct microbial cues, thereby modulating intestinal immune tone and tolerance. Simultaneously, secretory IgA enforces microbial containment and compositional balance, not only as an effector of humoral immunity but also as a mediator of immune education. Additional adaptive elements further expand this regulatory network, highlighting the systemic impact of microbial signals. This bidirectional communication ensures that immune responses are appropriately calibrated to preserve mucosal integrity while accommodating the vast antigenic diversity of the gut microbiome. Understanding these interactions provides a foundation for therapeutic strategies targeting dysbiosis-related diseases, including IBD, allergies, and autoimmunity.

## 3 Dysbiosis-immune axis in in intestinal diseases

**Figure 2:**
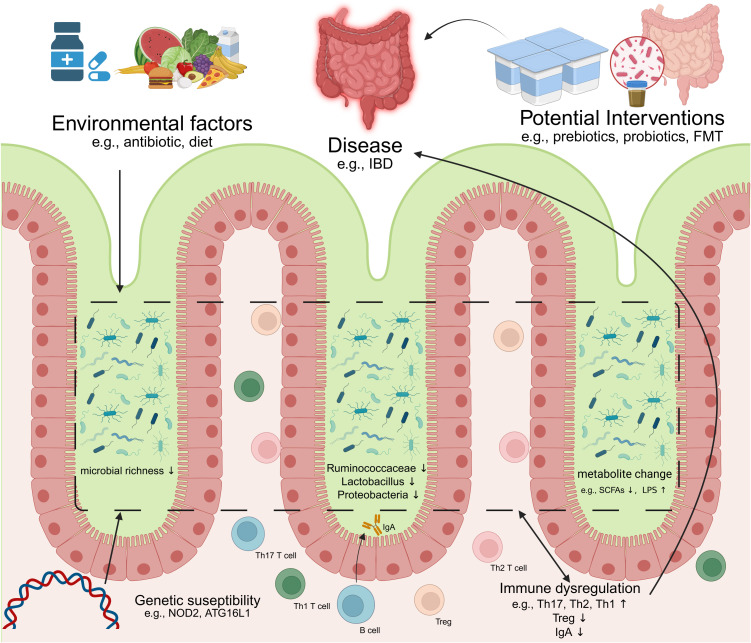
Dysregulation of microbiome-immunity interaction in disease. IBD, inflammatory bowel diseases; FMT, Fecal microbiota transplantation; Th17, T helper 17 cell; Th1, T helper 1 cell; Th2, T helper 2 cell; Treg, Regulatory T cell; SCFAs, Short-chain fatty acids; LPS, lipopolysaccharide(Created in https://BioRender.com).

Mounting evidence indicates that dysbiosis-driven immune dysregulation serves as a cornerstone in the pathogenesis of intestinal pathologies (**Figure 2**). In genetically susceptible hosts, compromised mucosal barrier integrity—characterized by disrupted tight junctions and increased permeability—permits microbial metabolite translocation, initiating a cascade of inflammatory responses (108). This breach of intestinal homeostasis synergizes with immune imbalances: attenuated Tregs suppression, aberrant B cell activation, and skewed Th1/Th17 polarization collectively fuel chronic inflammation (109, 110). Critically, such maladaptations transcend the gut, with molecular mimicry mechanisms (e.g., microbial antigen cross-reactivity with host epitopes) linking enteric dysbiosis to systemic autoimmunity (111). While causal relationships remain under investigation, the convergence of genetic vulnerability (e.g., NOD2 mutations), environmental triggers (e.g., antibiotics/diet), and microbiome alterations establishes a permissive milieu for disease onset and progression. Below we delineate how these interactions manifest in specific intestinal and extra-intestinal disorders. an imbalance of intestinal immunity related to Th2 cytokines, while CD is associate to a Th1 and Th17 cytokine profile (Heller et al., 2005). In CD, differentiation into Th1 and Th17 occurs by induction of cytokines IL-12, IL-18, IL-23 and transforming growth factor beta (TGFβ) produced by macrophages and other APC. In UC, increased secretion of IL-5, which is Th2 specific, is related to more effective activation of B cells and stimulation of immune responses when compared to the Th1 response observed in CD. Although the precise mechanisms underlying IBS remain unclear, it is widely recognized that its pathogenesis results from the interplay between genetic predisposition and environmental factors within the microbiome. This interaction, facilitated by a compromised intestinal epithelium, leads to excessive immune activation, which is thought to contribute to the clinical manifestations observed in IBD.

### 3.1 IBD: microbial triggers of immune dysregulation

IBD, encompassing CD and UC, represents chronic, relapsing-remitting inflammatory conditions of the gastrointestinal tract exhibiting escalating global incidence (112). Compelling evidence implicates gut microbiome perturbations—dysbiosis—as central to IBD pathogenesis (18). Characteristically, IBD patients typically exhibit reduced bacterial diversity, with notable shifts in the abundance of specific bacterial taxa (113–115). For example, the abundances of *Bacteroides*, *Firmicutes*, *Clostridia*, *Lactobacillus*, and *Ruminococcaceae* decrease, while those of *Gammaproteobacteria* and *Enterobacteriaceae* increase (116, 117). Concurrently, the profiles of microbiome-associated metabolites are altered (118, 119).

To elucidate the underlying mechanisms, we turn to the role of epithelial integrity and genetic susceptibility. A critical pathophysiological event involves the breakdown of tightly regulated intestinal barrier integrity. This breach facilitates translocation of commensal bacteria into the mucosal lamina propria, triggering aberrant host immune activation and subsequent tissue damage (120). Barrier defects encompass multiple components: compromised mucus layer architecture (e.g., Muc2 deficiency, which precipitates spontaneous colitis and early dysbiosis in susceptible murine models), impaired epithelial tight junctions, and dysregulated AMPs secretion (121).

These structural vulnerabilities are further exacerbated by genetic predispositions that affect microbial sensing and immune response. Genome-wide association studies have identified >200 IBD susceptibility loci, many encoding proteins critical for microbial immune sensing and response (122). The NOD2 (nucleotide-binding oligomerization domain-containing protein 2) mutation, the first strongly associated CD risk allele, exemplifies this link (123). As an intracellular PRR detecting bacterial peptidoglycan, NOD2 regulates commensal ecology by modulating AMPs expression and suppressing expansion of pro-inflammatory species like Bacteroides vulgatus (124, 125). Dysfunctional microbiome-immune crosstalk consequent to NOD2 mutation is thus pivotal in CD development (126). Similarly, mutations in autophagy-related 16-like 1 (ATG16L1), another CD-associated allele, impair Paneth cell exocytosis and exacerbate inflammatory responses and epithelial necrosis via dysregulated IL-22 signaling (127, 128). Inflammasome signaling further modulates this axis; NLRP6 inflammasome perturbation, for instance, heightens susceptibility to murine colitis and potentiates inflammation in IL10^-^/^-^ mice (129).

Beyond innate immunity, adaptive immune components significantly shape disease course. While the role of adaptive immunity—encompassing effector T cells, Tregs, and humoral responses—in expanding IBD-associated pathobionts is well-documented (130). establishing definitive causality between microbiome alterations and inflammation remains complex (111). Nonetheless, emerging evidence supports a contributory role for dysbiosis: Fecal microbiota transplantation from CD patients into germ-free mice harboring susceptibility genes triggers CD-like inflammation (131). Microbiota from IBD patients can also induce imbalances in intestinal Th17 and RORγt^+^ Tregs populations in germ-free recipients (132). Furthermore, specific pathobionts isolated from IBD patients, such as *Mucispirillum schaedleri* and adherent-invasive *Escherichia coli* (AIEC) strains, elicit colitis in susceptible murine models (133, 134).

In conclusion, dysregulated crosstalk between the gut microbiome and host immune system constitutes a fundamental mechanism underpinning IBD pathogenesis (**Table 1**). Future research must prioritize elucidating the causal directionality of these interactions to inform targeted therapeutic interventions.

**Table 1:** Dysbiosis-immune interactions in major intestinal diseases.

Disease	Key microbial alterations	Immune system impact	Underlying mechanisms & evidence	References
IBD	↓ Diversity, ↓ *Bacteroides*, ↓ *Firmicutes* (e.g., *Clostridium clusters* IV, XIVa), ↓ *Faecalibacterium prausnitzii* ↑ *Gammaproteobacteria* (e.g., AIEC)	↑ Th1/Th17 responses ↓ Treg function ↓ IgA coating	NOD2 mutations →↓ defensins→ bacterial translocation; AIEC activates IFN-γ/IL-23 axis	Okolie et al., 2024 (126)
			ATG16L1 mutations → impair Paneth cell exocytosis and disrupt IL-22 signaling → inflammatory responses and epithelial necrosis	Aden et al., 2018; Cadwell et al., 2009 (127, 128)
IBS	↓ Diversity, ↓ SCFA producers ↑ *Bacteroides*, ↑ *Escherichia*, ↑ *Clostridium* spp.	Mucosal immune activation (↑ IL-1β, TNF-α) Mast cell activation → Visceral hypersensitivity ↓ Treg function	Flagellin from *Clostridium* XIVa → TLR5 activation → epithelial NF-κB signaling	Jeffery et al., 2012 (149)
			Dietary factors (high-fat/high-sugar diets) →↓ SCFAs concentrations; pro-inflammatory bacteria → impairing epithelial barrier function	Agus et al., 2016; Vojdani et al., 2020 (159, 160)
CRC	↑ *Fusobacterium nucleatum* ↑ Enterotoxigenic *B. fragilis* (ETBF) ↑ *Akkermansia muciniphila* (context-dependent) ↓ *Ruminococcus gnavus*, ↓ *Blautia producta*	Suppressed NK & CD8^+^ T cell cytotoxicity ↑ Th17-driven inflammation ↑ Treg accumulation	↑ *Fusobacterium nucleatum* →↓NK cell cytotoxicity	Gur et al., 2015; Pignatelli et al., 2023 (163, 164)
			↑ *Fusobacterium nucleatum* →↓CD3^+^ T cells	Hamada et al., 2018 (165)
			↑ *Akkermansia muciniphila* → synergistic interactions with commensal or pathogenic bacteria → suppress or promote carcinogenesis	Gubernatorova et al., 2023 (167)
			BFT toxin → IL-17-dependent NF-κB pathway	Chung et al., 2018 (169)
			BFT toxin → E-cadherin cleavage → β-catenin activation	DeDecker et al., 2021 (171)

#### 3.1.1 Acute infectious insults as a model of dysbiosis-immune dysregulation

Acute gastrointestinal infections model how a breach in host-microbiota mutualism precipitates immune dysfunction. The intestinal epithelium is the primary barrier, fortified by the resident microbiota. GF mice and animals deficient in microbial sensing (e.g., Nod2^-^/^-^, MyD88^-^/^-^) exhibit impaired AMPs production, compromising barrier integrity and facilitating pathogen translocation (135, 136). Deficiencies in AMPs (e.g., RegIIIγ) lead to elevated mucosal bacterial colonization (73).

The IL-22-regulated AMPs axis is critical for defense against enteric pathogens (137–140). sIgA, whose expression is modulated by microbiota, binds antigens and neutralizes pathogens (141–143). Systemically, the microbiota primes IL-1β for neutrophil mobilization and stimulates TH17 cell expansion, contributing to pathogen resistance (144).

### 3.2 IBS: gut microbiota–immune interplay and low-grade inflammation

IBS is increasingly recognized as a disorder driven by microbial perturbations and characterized by subtle yet persistent immunological disturbances (145). Unlike IBD, IBS lacks macroscopic inflammation and is instead marked by low-grade immune activation. In post-infectious IBS (PI-IBS), this is often initiated by acute enteric infections that compromise epithelial integrity, allowing microbial components to activate mucosal immunity (146). Approximately 10% of individuals who experience acute enteritis develop IBS symptoms, highlighting the link between barrier disruption and immune sensitization.

This chronic immune stimulation is frequently associated with gut dysbiosis. IBS patients often present with gut microbiota dysbiosis, characterized by reduced bacterial diversity, a depletion of beneficial taxa (e.g., *Lactobacillus*, *Bifidobacterium*), and an enrichment of pathobionts like *Escherichia coli*, *Bacteroides*, and *Clostridium* species (147, 148). These alterations are not merely associative; many of the enriched bacterial taxa express immune-activating molecules such as lipopolysaccharide (LPS) and flagellin, which engage PRRs on intestinal epithelial and immune cells, particularly TLR4 and TLR5, driving mucosal immune activation and barrier dysfunction (149).

From innate activation to adaptive immune consequences, further complexity arises in the immunopathology of IBS Increased intestinal permeability following infection allows microbial antigens to reach the lamina propria, where they stimulate dendritic cells, macrophages, and mast cells, triggering Th1 and Th17 differentiation (150–152). Elevated levels of pro-inflammatory cytokines—such as interleukin-1β (IL-1β), tumor necrosis factor-alpha (TNF-α), and interferon-gamma (IFN-γ)—are frequently observed, while anti-inflammatory mediators (e.g., IL-10, IL-13) are often suppressed (153, 154).

Microbial metabolites, particularly SCFAs such as butyrate, acetate, and propionate, play a pivotal role in modulating intestinal immunity and maintaining mucosal homeostasis (155). Produced through bacterial fermentation of dietary fibers, SCFAs exert their immunoregulatory effects by promoting the differentiation of Tregs via histone deacetylase inhibition, activating G-protein-coupled receptors (GPR43, GPR109A), and enhancing epithelial barrier integrity (40–42). However, SCFAs production is diminished in IBS patients due to dietary factors and depletion of key producers such as *Faecalibacterium prausnitzii* (156). Concurrently, other bacterial metabolites (e.g., histamine, 5-HT, dopamine) can directly modulate sensory nerve function, further amplifying nociceptive signaling (157, 158).

Lastly, we consider how environmental factors amplify this dysbiosis-immune feedback loop. Dietary patterns (e.g., high-fat, low-fiber intake) and psychological stress activate the hypothalamic-pituitary-adrenal (HPA) axis, releasing corticotropin-releasing hormone (CRH) that triggers mast cell activation and barrier dysfunction (159, 160). These changes perpetuate a vicious cycle wherein immune activation reshapes microbial niches, worsening dysbiosis.

In summary, IBS is increasingly recognized as an immunological disorder driven by microbial perturbations. The convergence of low-grade inflammation, dysregulated metabolite signaling, and neuroimmune sensitization establishes a new paradigm for understanding IBS and guiding microbiota-targeted therapies.

### 3.3 CRC: microbiota-mediated immune evasion and carcinogenesis

The gut microbiota critically modulates cancer immune surveillance through dynamic interactions with host immunity (18). CRC is the most common cancer of the digestive system with high mortality and morbidity rates (161). Compared to healthy individuals, patients with CRC exhibit reduced gut microbial diversity and distinct dysbiosis (162). Within the colorectal tumor microenvironment (TME), specific bacterial species actively impair antitumor immunity. A prominent example is *Fusobacterium nucleatum*, which accumulates in CRC tissues and directly inhibits NK cell cytotoxicity. This immunosuppressive effect is primarily mediated by the binding of the bacterial Fap2 protein to the inhibitory receptor TIGIT on NK cells (163, 164). Clinically, elevated abundance of *F. nucleatum* in human CRC correlates with reduced intratumoral infiltration of CD3^+^ T cells—a lymphocyte population associated with improved patient survival—further implicating this bacterium in promoting an immunosuppressive TME (165).

Furthermore, increased abundance of *Akkermansia muciniphila* in patients with epithelial tumors correlates positively with response to PD-1 blockade. This effect potentially involves the recruitment of CCR9^+^CXCR3^+^CD4^+^ T lymphocytes to tumors and enhanced IL-12 secretion (166). Research on *A. muciniphila* in CRC reveals context-dependent outcomes. Murine studies demonstrate conflicting roles: while some report protective effects, others show administration exacerbates tumorigenesis. Emerging consensus attributes these discrepancies to strain-specific properties, as *A. muciniphila* may degrade the mucin barrier and engage in synergistic interactions with commensal or pathogenic bacteria that either suppress or promote carcinogenesis (167).

Similarly, enterotoxigenic *B. fragilis* (BFT^+^) contributes to early carcinogenesis. Its secreted metalloproteinase toxin (BFT) is implicated in human colonic adenoma and serrated polyp formation, promotes Th17-mediated colitis in mouse models (168), and drives distal CRC in Apcmin+/− mice via IL-17-dependent NF-κB pathway activation (169). BFT^+^*B. fragilis* colonization additionally facilitates regulatory T cell accumulation, inducing IL-17-driven procarcinogenic inflammation (170). At the epithelial level, BFT cleaves E-cadherin, increasing paracellular permeability and activating β-catenin signaling to enhance proliferation (171). It further induces DNA damage through polyamine catabolism in CRC cells (172). Notably, this bacterium promotes local dysbiosis by expanding other procarcinogenic species, compromising host immunity (170), disrupting the gut barrier (171), and degrading mucin (173).

In contrast, commensals like *Ruminococcus gnavus* and *Blautia producta* enhance antitumor immunity. They degrade lysoglycerophospholipids within the intestinal niche, thereby potentiating the tumor immune surveillance function of CD8^+^ T cells and inhibiting colon carcinogenesis (174).

## 4 Conclusion and future perspectives

The intricate bidirectional interplay between the gut microbiome and the host immune system is a cornerstone of intestinal health and a key factor in the pathogenesis of a spectrum of diseases. While this review has synthesized compelling evidence of how dysbiosis drives immune dysfunction in IBD, IBS, CRC, and following infection, it is crucial to recognize the significant limitations inherent in this rapidly evolving field. A primary challenge remains establishing definitive causality rather than correlation. While animal models, particularly gnotobiotic mice, have been indispensable for mechanistic dissection, they often fall short of recapitulating the full complexity of human physiology, genetics, and environmental exposures. The widely used inbred laboratory mice possess a depauperate microbiota and an immune system calibrated for this simplified community, which may yield exaggerated or misleading effects compared to humans harboring a far more complex and resilient microbial ecosystem. Furthermore, the staggering inter-individual heterogeneity in both microbiome composition and immune responses often exceeds differences related to disease status itself, complicating the identification of universal therapeutic targets and the translation of findings from population-level studies to the individual patient.

To overcome these hurdles and move from association to mechanism, future research must embrace a multi-faceted and integrative approach:

Multi-omics Integration: Disentangling causality will require the longitudinal collection and integrated analysis of multi-omics datasets—encompassing metagenomics, metatranscriptomics, metabolomics, metaproteomics, and host epigenomics—from well-characterized human cohorts. This will help bridge the gap between microbial taxonomy, gene expression, functional output, and host response, revealing the active drivers of immune modulation.

Next-Generation Animal Models: The field must transition beyond conventional laboratory mice. “Wildling” or “dirty” mouse models, which are colonized with a complex, naturalized microbiota from wild mice or human donors, offer a more physiologically relevant preclinical platform. These models exhibit immune responses closer to humans and can better predict the efficacy and safety of microbiota-targeted interventions.

Leveraging Artificial Intelligence: Machine learning and artificial intelligence (AI) are poised to play a transformative role. These tools can decipher the immense complexity of multi-omics data, identify predictive biomarkers of disease susceptibility or treatment response, and ultimately build models for personalized microbiome medicine. AI can help navigate the heterogeneity problem by stratifying patients into subpopulations based on their unique microbiome-immune signatures.

Expanding the Microbiome Definition: Future studies must look beyond bacteria to fully incorporate the virome (phages), mycobiome (fungi), and archaea into the ecosystem-level understanding of host-microbe interactions. Their roles in modulating immune tone and influencing bacterial community dynamics are still poorly understood but are likely significant.

The therapeutic landscape targeting the microbiota-immune axis is promising yet fraught with challenges. While Fecal Microbiota Transplantation (FMT) has demonstrated proof-of-principle, its long-term safety, variable efficacy, and lack of standardized protocols necessitate caution. Next-generation live biotherapeutic products (LBPs) and precision prebiotics offer a more controlled approach but must demonstrate robust and reproducible effects in diverse human populations. Similarly, while postbiotic strategies (e.g., administering SCFAs) are attractive for their safety and defined nature, their efficacy may be limited by the redundancy and complexity of microbial metabolic networks *in vivo*.

In conclusion, while the past decade has yielded profound insights into the microbiota-immune dialogue, the path forward requires a critical acknowledgment of current limitations and a concerted effort to adopt more sophisticated, integrative, and human-relevant approaches. By harnessing the power of multi-omics, advanced animal models, and computational biology, the field can move beyond descriptive associations and toward a causal, mechanistic, and ultimately translational understanding of how to harness the microbiome for immune health. This refined knowledge is essential for developing the next generation of safe, effective, and personalized therapies for the multitude of diseases rooted in a disrupted microbiota-immune equilibrium.
